# 5-(Trifluoro­meth­oxy)isatin

**DOI:** 10.1107/S1600536812044297

**Published:** 2012-11-28

**Authors:** M. Schutte, C. Pretorius, H.G. Visser, A. Roodt

**Affiliations:** a Department of Chemistry, University of the Free State, PO Box 339, Bloemfontein, 9301, South Africa

## Abstract

The title compound [systematic name: 5-(trifluoro­meth­oxy)-1*H*-indole-2,3-dione], C_9_H_4_F_3_NO_3_, crystallized with two mol­ecules in the asymmetric unit. Inter­molecular N—H⋯O hydrogen bonds link the mol­ecules to form layers parallel to the *ab* plane. In addition, π–π stacking inter­actions are observed with a centroid–centroid distance of 3.721 (1) Å. The near planarity of the two isatin ring systems is illustrated by by the maximum deviations of 0.023 (1) and 0.025 (1) Å for the N atom in each case.

## Related literature

For similar structures and background information on isatin as a biological agent, see Schutte *et al.* (2012 ▶); Garden *et al.* (2006 ▶); Goldschmidt & Llewellyn (1950 ▶); Frolova *et al.* (1988 ▶); Wei *et al.* (2004 ▶); Palmer *et al.* (1987 ▶); Akkurt *et al.* (2006 ▶). For reaction kinetic data on similar structures, see: Schutte *et al.* (2011 ▶).
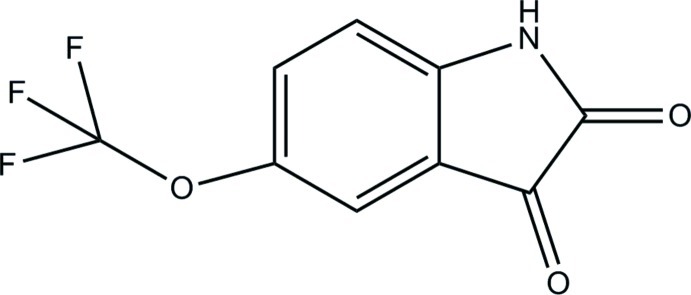


## Experimental

### 

#### Crystal data

C_9_H_4_F_3_NO_3_
*M* *_r_* = 231.13
Monoclinic, 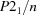

*a* = 14.329 (5) Å
*b* = 6.082 (5) Å
*c* = 20.401 (5) Å
β = 91.608 (5)°
*V* = 1777.2 (16) Å^3^
*Z* = 8
Mo *K*α radiation
μ = 0.17 mm^−1^
*T* = 100 K
0.51 × 0.18 × 0.10 mm

#### Data collection

Bruker APEXII CCD diffractometer
Absorption correction: multi-scan (*SADABS*; Bruker, 2008 ▶) *T*_min_ = 0.152, *T*_max_ = 0.394
26198 measured reflections
4428 independent reflections
3513 reflections with *I* > 2σ(*I*)
*R*_int_ = 0.029

#### Refinement

*R*[*F* ^2^ > 2σ(*F* ^2^)] = 0.036
*wR*(*F* ^2^) = 0.102
*S* = 1.04
4418 reflections
297 parameters
H atoms treated by a mixture of independent and constrained refinement
Δρ_max_ = 0.34 e Å^−3^
Δρ_min_ = −0.33 e Å^−3^

### 

Data collection: *APEX2* (Bruker, 2008 ▶); cell refinement: *SAINT-Plus* (Bruker, 2008 ▶); data reduction: *SAINT-Plus*; program(s) used to solve structure: *SHELXS97* (Sheldrick, 2008 ▶); program(s) used to refine structure: *SHELXL97* (Sheldrick, 2008 ▶); molecular graphics: *DIAMOND* (Brandenburg & Putz, 2005 ▶); software used to prepare material for publication: *WinGX* (Farrugia, 1999 ▶).

## Supplementary Material

Crystal structure: contains datablock(s) global, I. DOI: 10.1107/S1600536812044297/fy2072sup1.cif

Structure factors: contains datablock(s) I. DOI: 10.1107/S1600536812044297/fy2072Isup2.hkl

Supplementary material file. DOI: 10.1107/S1600536812044297/fy2072Isup3.cml

Additional supplementary materials:  crystallographic information; 3D view; checkCIF report

## Figures and Tables

**Table 1:** Hydrogen-bond geometry (Å, °)

*D*—H⋯*A*	*D*—H	H⋯*A*	*D*⋯*A*	*D*—H⋯*A*
N1—H1⋯O4^i^	0.84 (2)	1.99 (2)	2.7615 (18)	152.8 (18)
N2—H2⋯O1^ii^	0.89 (2)	2.03 (2)	2.8776 (18)	157.4 (19)
N2—H2⋯O4^iii^	0.89 (2)	2.55 (2)	2.9850 (18)	111.2 (16)
C16—H16⋯F3^i^	0.93	2.39	3.171 (2)	142
C18—H18⋯O2^iv^	0.93	2.47	3.327 (3)	153

Symmetry codes: (i) 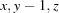; (ii) 
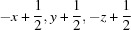
; (iii) 
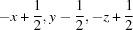
; (iv) 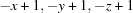.

**Fig. 1.**
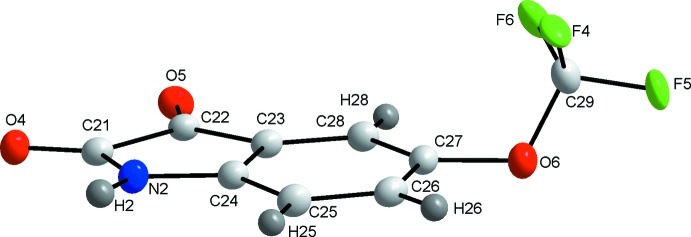
Representation of the title compound, showing the numbering scheme and displacement ellipsoids (50% probability).

**Fig. 2.**
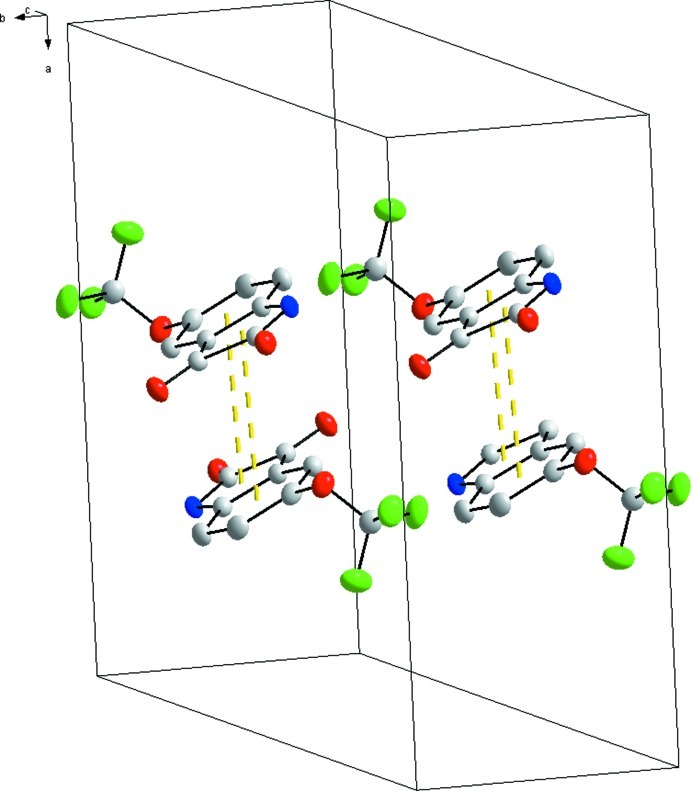
Observed π-π stacking (indicated by dashed lines) and packing in the crystal structure (hydrogen atoms omitted for clarity).

## supplementary crystallographic information

### Comment

5-(Trifluoromethoxy)isatin crystallized in the *P*2_1_/n spacegroup and with two independent molecules in the asymmetric unit. The use of 5-(trifluormethoxy)isatin forms part of a current study, where water soluble O,*O*'-donor bidentate ligands are coordinated to the rhenium tricarbonyl core in order to study the reactivity and activation of these potential radiopharmaceutical compounds. Previous studies have shown that the O,*O*'-bidenate ligands activate the Re(I) core to a great extent and we would like to explore this further (Schutte *et al.* (2011). The isatin molecule also has a variety of biological activities. It can act as an anticonvulsant agent, it has sedative effects but can also cause anxiety. Palmer *et al.*, 1987; Goldschmidt & Llewellyn, 1950; Wei *et al.*, 2004; Frolova *et al.*, 1988; Akkurt *et al.*, 2006). Due to its diverse pharmacological properties, a lot of interest has been shown in the isatin molecule and its derivatives. All bond lengths and angles compare well to the structure of *N*-benzylisatin reported by Schutte *et al.* (2012). Five intermolecular hydrogen interactions are observed in the structure (C—H···O, N—H···O and C—H···F) as well as π-π stacking with a centroid to centroid distance of 3.721 (1) Å. The molecules pack in a head to head fashion in alternating layers with the benzyl ring overlapping with the next molecules' pyrrolidinedione ring and *vice versa*. The π-π stacking and the packing in the unit cell are illustrated in Figure 2. The planarity of the ring systems, N1—C11—C12—C13—C14—C15—C16—C17—C18 and N2—C21—C22—C23—C24—C25—C26—C27—C28, are illustrated by very small deviations of all the atoms from these planes, with the largest deviations 0.023 (1) Å for N1 and 0.025 (1) for N2 respectively.

### Experimental

5-(Trifluoromethoxy)isatin was dissolved in water (pH 4). The crystls were grown from a vapour diffusion setup with chloroform at 5 °C in a commercial refrigerator.

### Refinement

Aromatic H atoms were positioned geometrically and allowed to ride on their parent atoms, with *U*_iso_(H) = 1.2 *U*_eq_(parent) and with a C—H distance of 0.93 Å. N-bound hydrogen atoms were placed from the electron density map and refined freely. 10 reflections with large negative intensities [I < –4σ(I)] were excluded from the refinement.

### Crystal data

**Table:**

C_9_H_4_F_3_NO_3_	*F*(000) = 928
*M**_r_* = 231.13	*D*_x_ = 1.728 Mg m^−^^3^
Monoclinic, *P*2_1_/*n*	Mo *K*α radiation, λ = 0.71069 Å
*a* = 14.329 (5) Å	Cell parameters from 9825 reflections
*b* = 6.082 (5) Å	θ = 2.8–28.2°
*c* = 20.401 (5) Å	µ = 0.17 mm^−^^1^
β = 91.608 (5)°	*T* = 100 K
*V* = 1777.2 (16) Å^3^	Needle, orange
*Z* = 8	0.51 × 0.18 × 0.10 mm

### Data collection

**Table:**

Bruker APEXII CCD diffractometer	3513 reflections with *I* > 2σ(*I*)
Graphite monochromator	*R*_int_ = 0.029
φ and ω scans	θ_max_ = 28.3°, θ_min_ = 1.8°
Absorption correction: multi-scan (*SADABS*; Bruker, 2008)	*h* = −19→19
*T*_min_ = 0.152, *T*_max_ = 0.394	*k* = −7→8
26198 measured reflections	*l* = −27→27
4428 independent reflections	

### Refinement

**Table:**

Refinement on *F*^2^	Primary atom site location: structure-invariant direct methods
Least-squares matrix: full	Secondary atom site location: difference Fourier map
*R*[*F*^2^ > 2σ(*F*^2^)] = 0.036	Hydrogen site location: inferred from neighbouring sites
*wR*(*F*^2^) = 0.102	H atoms treated by a mixture of independent and constrained refinement
*S* = 1.04	*w* = 1/[σ^2^(*F*_o_^2^) + (0.0458*P*)^2^ + 0.9713*P*] where *P* = (*F*_o_^2^ + 2*F*_c_^2^)/3
4418 reflections	(Δ/σ)_max_ = 0.001
297 parameters	Δρ_max_ = 0.34 e Å^−^^3^
0 restraints	Δρ_min_ = −0.33 e Å^−^^3^

### Special details

**Table:**

Geometry. All s.u.'s (except the s.u. in the dihedral angle between two l.s. planes) are estimated using the full covariance matrix. The cell s.u.'s are taken into account individually in the estimation of s.u.'s in distances, angles and torsion angles; correlations between s.u.'s in cell parameters are only used when they are defined by crystal symmetry. An approximate (isotropic) treatment of cell s.u.'s is used for estimating s.u.'s involving l.s. planes.
Refinement. Refinement of *F*^2^ against ALL reflections. The weighted *R*-factor *wR* and goodness of fit *S* are based on *F*^2^, conventional *R*-factors *R* are based on *F*, with *F* set to zero for negative *F*^2^. The threshold expression of *F*^2^ > 2σ(*F*^2^) is used only for calculating *R*-factors(gt) *etc*. and is not relevant to the choice of reflections for refinement. *R*-factors based on *F*^2^ are statistically about twice as large as those based on *F*, and *R*- factors based on ALL data will be even larger.

### Fractional atomic coordinates and isotropic or equivalent isotropic displacement parameters (Å^2^)

**Table:**

	*x*	*y*	*z*	*U*_iso_*/*U*_eq_	
O2	0.44882 (8)	0.39123 (17)	0.43829 (5)	0.0258 (2)	
O4	0.25592 (7)	0.62417 (16)	0.33404 (5)	0.0222 (2)	
O3	0.41251 (8)	0.06479 (18)	0.69260 (5)	0.0255 (2)	
O6	0.01035 (8)	−0.39384 (17)	0.38480 (5)	0.0258 (2)	
N1	0.33820 (9)	−0.1165 (2)	0.43057 (6)	0.0207 (3)	
F3	0.35956 (8)	0.40475 (15)	0.70118 (5)	0.0375 (3)	
O5	0.21790 (7)	0.35661 (17)	0.44843 (5)	0.0230 (2)	
F2	0.38339 (8)	0.20705 (17)	0.78654 (5)	0.0366 (2)	
F5	−0.09269 (8)	−0.57440 (18)	0.43734 (6)	0.0482 (3)	
O1	0.37037 (8)	0.09137 (17)	0.33911 (5)	0.0241 (2)	
N2	0.16708 (9)	0.3571 (2)	0.28133 (6)	0.0202 (3)	
C13	0.39620 (10)	0.1071 (2)	0.51369 (7)	0.0188 (3)	
C14	0.34985 (10)	−0.0899 (2)	0.49875 (7)	0.0196 (3)	
C11	0.37229 (10)	0.0574 (2)	0.39792 (7)	0.0202 (3)	
C23	0.13214 (10)	0.1310 (2)	0.36813 (7)	0.0184 (3)	
F6	−0.05978 (9)	−0.2481 (2)	0.46963 (6)	0.0564 (4)	
C18	0.41720 (10)	0.1665 (2)	0.57812 (7)	0.0202 (3)	
H18	0.449	0.2958	0.5885	0.024*	
F1	0.26737 (7)	0.1395 (2)	0.72116 (5)	0.0436 (3)	
F4	−0.13875 (8)	−0.2999 (2)	0.38080 (7)	0.0564 (4)	
C15	0.32098 (11)	−0.2309 (2)	0.54708 (8)	0.0244 (3)	
H15	0.2897	−0.361	0.5369	0.029*	
C17	0.38837 (10)	0.0232 (2)	0.62607 (7)	0.0217 (3)	
C26	0.03648 (10)	−0.1692 (2)	0.29098 (7)	0.0221 (3)	
H26	0.0038	−0.2727	0.2659	0.027*	
C24	0.12267 (10)	0.1618 (2)	0.30027 (7)	0.0190 (3)	
C21	0.20969 (10)	0.4565 (2)	0.33289 (7)	0.0195 (3)	
C28	0.09416 (10)	−0.0507 (2)	0.39831 (7)	0.0198 (3)	
H28	0.101	−0.0734	0.4433	0.024*	
C27	0.04544 (10)	−0.1968 (2)	0.35822 (7)	0.0209 (3)	
C12	0.41347 (10)	0.2177 (2)	0.45094 (7)	0.0196 (3)	
C29	−0.06921 (12)	−0.3765 (3)	0.41759 (9)	0.0324 (4)	
C22	0.18838 (10)	0.3137 (2)	0.39389 (7)	0.0182 (3)	
C25	0.07594 (10)	0.0119 (2)	0.26051 (7)	0.0217 (3)	
H25	0.071	0.0311	0.2153	0.026*	
C19	0.35582 (11)	0.2012 (3)	0.72439 (7)	0.0251 (3)	
C16	0.34070 (11)	−0.1702 (2)	0.61182 (8)	0.0250 (3)	
H16	0.3217	−0.2602	0.6458	0.03*	
H1	0.3123 (13)	−0.224 (3)	0.4120 (9)	0.028 (5)*	
H2	0.1668 (14)	0.403 (3)	0.2399 (11)	0.041 (6)*	

### Atomic displacement parameters (Å^2^)

**Table:**

	*U*^11^	*U*^22^	*U*^33^	*U*^12^	*U*^13^	*U*^23^
O2	0.0326 (6)	0.0228 (5)	0.0219 (5)	−0.0080 (4)	−0.0022 (4)	0.0025 (4)
O4	0.0266 (5)	0.0198 (5)	0.0204 (5)	−0.0040 (4)	0.0004 (4)	−0.0013 (4)
O3	0.0346 (6)	0.0267 (5)	0.0150 (5)	0.0069 (4)	−0.0031 (4)	0.0020 (4)
O6	0.0279 (6)	0.0206 (5)	0.0291 (6)	−0.0038 (4)	0.0033 (4)	0.0012 (4)
N1	0.0249 (6)	0.0178 (6)	0.0193 (6)	−0.0031 (5)	−0.0038 (5)	−0.0016 (5)
F3	0.0583 (7)	0.0238 (5)	0.0308 (5)	0.0088 (4)	0.0079 (5)	0.0058 (4)
O5	0.0240 (5)	0.0285 (5)	0.0165 (5)	−0.0019 (4)	−0.0021 (4)	−0.0034 (4)
F2	0.0518 (6)	0.0408 (6)	0.0171 (5)	0.0045 (5)	0.0000 (4)	0.0003 (4)
F5	0.0507 (7)	0.0394 (6)	0.0552 (7)	−0.0198 (5)	0.0141 (6)	0.0074 (5)
O1	0.0310 (6)	0.0246 (5)	0.0166 (5)	−0.0014 (4)	−0.0024 (4)	0.0000 (4)
N2	0.0259 (6)	0.0198 (6)	0.0147 (6)	−0.0021 (5)	−0.0011 (5)	−0.0005 (5)
C13	0.0205 (7)	0.0176 (6)	0.0183 (7)	0.0003 (5)	−0.0016 (5)	0.0016 (5)
C14	0.0205 (7)	0.0189 (6)	0.0192 (7)	0.0006 (5)	−0.0022 (5)	0.0001 (5)
C11	0.0207 (7)	0.0195 (6)	0.0203 (7)	0.0005 (5)	−0.0020 (5)	−0.0012 (5)
C23	0.0194 (7)	0.0192 (6)	0.0165 (7)	0.0011 (5)	−0.0008 (5)	−0.0029 (5)
F6	0.0623 (8)	0.0617 (8)	0.0470 (7)	−0.0306 (6)	0.0310 (6)	−0.0241 (6)
C18	0.0219 (7)	0.0194 (6)	0.0192 (7)	0.0003 (5)	−0.0023 (5)	0.0002 (5)
F1	0.0322 (6)	0.0599 (7)	0.0391 (6)	−0.0086 (5)	0.0089 (5)	−0.0075 (5)
F4	0.0260 (6)	0.0664 (8)	0.0766 (9)	0.0006 (5)	0.0004 (6)	0.0168 (7)
C15	0.0283 (8)	0.0179 (7)	0.0269 (8)	−0.0025 (6)	−0.0004 (6)	0.0020 (6)
C17	0.0264 (7)	0.0229 (7)	0.0157 (7)	0.0047 (6)	−0.0018 (5)	0.0015 (5)
C26	0.0215 (7)	0.0206 (7)	0.0241 (7)	0.0003 (5)	−0.0013 (6)	−0.0057 (6)
C24	0.0199 (7)	0.0187 (6)	0.0183 (7)	0.0017 (5)	0.0000 (5)	−0.0006 (5)
C21	0.0207 (7)	0.0195 (6)	0.0182 (7)	0.0018 (5)	0.0007 (5)	−0.0020 (5)
C28	0.0211 (7)	0.0210 (7)	0.0175 (7)	0.0009 (5)	0.0002 (5)	−0.0010 (5)
C27	0.0203 (7)	0.0178 (6)	0.0247 (7)	−0.0001 (5)	0.0028 (6)	−0.0006 (5)
C12	0.0201 (7)	0.0204 (7)	0.0181 (7)	−0.0004 (5)	−0.0027 (5)	−0.0001 (5)
C29	0.0317 (9)	0.0316 (8)	0.0340 (9)	−0.0095 (7)	0.0050 (7)	−0.0019 (7)
C22	0.0183 (6)	0.0189 (6)	0.0173 (7)	0.0019 (5)	0.0008 (5)	−0.0019 (5)
C25	0.0240 (7)	0.0232 (7)	0.0178 (7)	0.0000 (6)	−0.0023 (5)	−0.0027 (5)
C19	0.0300 (8)	0.0260 (7)	0.0193 (7)	0.0010 (6)	0.0015 (6)	0.0043 (6)
C16	0.0316 (8)	0.0203 (7)	0.0233 (8)	0.0015 (6)	0.0028 (6)	0.0069 (6)

### Geometric parameters (Å, º)

**Table:**

O2—C12	1.2017 (19)	C11—C12	1.560 (2)
O4—C21	1.2157 (19)	C23—C28	1.384 (2)
O3—C19	1.3410 (19)	C23—C24	1.400 (2)
O3—C17	1.4140 (18)	C23—C22	1.462 (2)
O6—C29	1.342 (2)	F6—C29	1.322 (2)
O6—C27	1.4134 (19)	C18—C17	1.382 (2)
N1—C11	1.349 (2)	C18—H18	0.93
N1—C14	1.4055 (19)	F1—C19	1.322 (2)
N1—H1	0.84 (2)	F4—C29	1.316 (2)
F3—C19	1.327 (2)	C15—C16	1.393 (2)
O5—C22	1.2078 (17)	C15—H15	0.93
F2—C19	1.3180 (18)	C17—C16	1.387 (2)
F5—C29	1.316 (2)	C26—C27	1.384 (2)
O1—C11	1.2170 (18)	C26—C25	1.393 (2)
N2—C21	1.3450 (18)	C26—H26	0.93
N2—C24	1.407 (2)	C24—C25	1.381 (2)
N2—H2	0.89 (2)	C21—C22	1.555 (2)
C13—C18	1.388 (2)	C28—C27	1.383 (2)
C13—C14	1.399 (2)	C28—H28	0.93
C13—C12	1.473 (2)	C25—H25	0.93
C14—C15	1.379 (2)	C16—H16	0.93
			
C19—O3—C17	116.08 (12)	C23—C24—N2	110.67 (12)
C29—O6—C27	116.17 (12)	O4—C21—N2	128.85 (14)
C11—N1—C14	111.35 (12)	O4—C21—C22	124.91 (13)
C11—N1—H1	123.4 (13)	N2—C21—C22	106.24 (12)
C14—N1—H1	125.2 (13)	C27—C28—C23	116.62 (13)
C21—N2—C24	111.32 (12)	C27—C28—H28	121.7
C21—N2—H2	126.2 (14)	C23—C28—H28	121.7
C24—N2—H2	122.4 (14)	C28—C27—C26	122.61 (14)
C18—C13—C14	121.22 (13)	C28—C27—O6	119.79 (13)
C18—C13—C12	131.81 (13)	C26—C27—O6	117.33 (13)
C14—C13—C12	106.98 (12)	O2—C12—C13	132.05 (13)
C15—C14—C13	121.78 (14)	O2—C12—C11	123.61 (13)
C15—C14—N1	127.34 (14)	C13—C12—C11	104.33 (12)
C13—C14—N1	110.88 (12)	F5—C29—F4	107.62 (14)
O1—C11—N1	128.44 (13)	F5—C29—F6	108.41 (15)
O1—C11—C12	125.11 (13)	F4—C29—F6	107.90 (16)
N1—C11—C12	106.43 (12)	F5—C29—O6	108.00 (14)
C28—C23—C24	121.25 (13)	F4—C29—O6	112.56 (15)
C28—C23—C22	131.81 (13)	F6—C29—O6	112.18 (14)
C24—C23—C22	106.91 (12)	O5—C22—C23	132.15 (13)
C17—C18—C13	116.42 (13)	O5—C22—C21	123.01 (13)
C17—C18—H18	121.8	C23—C22—C21	104.81 (12)
C13—C18—H18	121.8	C24—C25—C26	117.14 (14)
C14—C15—C16	117.14 (14)	C24—C25—H25	121.4
C14—C15—H15	121.4	C26—C25—H25	121.4
C16—C15—H15	121.4	F2—C19—F1	108.46 (13)
C18—C17—C16	122.79 (14)	F2—C19—F3	107.71 (13)
C18—C17—O3	119.79 (13)	F1—C19—F3	107.18 (13)
C16—C17—O3	117.31 (13)	F2—C19—O3	108.22 (13)
C27—C26—C25	120.73 (13)	F1—C19—O3	113.15 (14)
C27—C26—H26	119.6	F3—C19—O3	111.94 (13)
C25—C26—H26	119.6	C17—C16—C15	120.63 (14)
C25—C24—C23	121.63 (13)	C17—C16—H16	119.7
C25—C24—N2	127.70 (13)	C15—C16—H16	119.7

### Hydrogen-bond geometry (Å, º)

**Table:**

*D*—H···*A*	*D*—H	H···*A*	*D*···*A*	*D*—H···*A*
N1—H1···O4^i^	0.84 (2)	1.99 (2)	2.7615 (18)	152.8 (18)
N2—H2···O1^ii^	0.89 (2)	2.03 (2)	2.8776 (18)	157.4 (19)
N2—H2···O4^iii^	0.89 (2)	2.55 (2)	2.9850 (18)	111.2 (16)
C16—H16···F3^i^	0.93	2.39	3.171 (2)	142
C18—H18···O2^iv^	0.93	2.47	3.327 (3)	153

Symmetry codes: (i) *x*, *y*−1, *z*; (ii) −*x*+1/2, *y*+1/2, −*z*+1/2; (iii) −*x*+1/2, *y*−1/2, −*z*+1/2; (iv) −*x*+1, −*y*+1, −*z*+1.
